# Measuring Online Teaching Service Quality in Higher Education in the COVID-19 Environment

**DOI:** 10.3390/ijerph18052403

**Published:** 2021-03-01

**Authors:** José M. Ramírez-Hurtado, Alfredo G. Hernández-Díaz, Ana D. López-Sánchez, Víctor E. Pérez-León

**Affiliations:** 1 Department of Economics, Quantitative Methods and Economy History, Faculty of Business, Pablo de Olavide University, Ctra. de Utrera, Km. 1, 41013 Seville, Spain; agarher@upo.es (A.G.H.-D.); adlopsan@upo.es (A.D.L.-S.); 2 Department of Applied Economics II, Faculty of Economics and Business, University of Seville, Avenida Ramón y Cajal, 1, 41018 Seville, Spain; vpleon@us.es

**Keywords:** service quality, higher education, online teaching, importance-performance analysis, structural equations

## Abstract

The use of the Internet to develop new technologies has generated a considerable change in teaching and student learning in higher education. The pandemic caused by COVID-19 has forced universities to switch from face-to-face to online instruction. Furthermore, this transfer process was planned and executed quickly, with urgent redesigns of courses originally conceived for live teaching. The aim of this work is to measure the service quality of online teaching delivered during the COVID-19 period. The methodology was based on an importance-performance analysis using a structural equations model. The data were obtained from a sample of 467 students attending a university in southern Spain. The results reveal five priority attributes of online teaching that need to be improved in order to enhance the service quality of the virtual instruction provided to students. Universities need to redefine their online format by integrating methodological and technological decisions and involving collaboration between teachers, students and administration staff and services. The results do not apply to educational institutions that exclusively teach courses online, but to those institutions that had to rapidly adapt, and shift course material originally designed for face-to-face training.

## 1. Introduction

The higher education sector is currently facing new challenges that had never previously experienced, arising from sharper global competitiveness, advances in technology and the rise in the numbers of universities that offer students an ever-broader range of courses to choose from. This has led universities and higher education centers to ponder how they can improve the service quality they offer their students. The use of Internet in education, especially in higher education, has generated considerable change in the way in which learning takes place. The e-learning model for teaching and learning has rapidly developed in recent years, mainly driven by technological advances [1]. E-learning spans a wide range of methods, applications, processes and academic areas, making difficult to agree on a commonly accepted definition of what e-learning is [2]. With the substantial growth in demand for e-learning, further research is needed on the factors that influence the adoption of this form of instruction [3].

Due to COVID-19, there was a rapid decline in face-to-face teaching in early 2020, with the closure of campuses, schools and other education centers, which saw on-site teaching shift rapidly online. According to the United Nations Educational, Scientific and Cultural Organization (UNESCO), more than 1.5 billion students in 165 countries have been affected by the closure of education centers, impacting around 90% of the world student population [4]; and not only students are affected: the shutdown has hit teachers and families, and will have long-term economic and social consequences. The type of online teaching that has taken place in recent months cannot be compared in terms of experience, planning and development to the proposals specifically developed for online teaching in its original pre-pandemic form [5]. Since the pandemic emerged, teachers have had to redesign and plan how they teach subjects that were originally conceived for the classroom. An important drawback is that not all teachers and students possess the technological media or digital competences required for giving and receiving virtual classes. This new situation has emphasized the significant digital divide among both teachers and students [6]. In this sense, institutions need to provide more suitable platforms for online teaching [7]. Despite this, the drive towards online teaching worldwide gathers pace.

The COVID-19 crisis has presented an opportunity for the development of effective learning solutions [8]. Universities anchored to the traditional face-to-face teaching model have striven to adopt strategies to ensure the service quality of their online teaching to satisfy their clients, namely the students. This makes research on the service quality of virtual training more important than ever. Students’ perceptions of e-learning are a crucial indicator of the quality of the learning experience and the results obtained, and studies of student satisfaction with e-learning abound [9]. The study of service quality in e-learning is vital to ensure that the implementation of any online learning system meets students’ learning needs. Service quality measurement models were first developed in the industrial and manufacturing sectors, but they cannot be applied directly to higher education. Many studies have highlighted the differences, hence the need for new service quality models that apply strictly to online teaching [10,11]. As it stands, there is no standard model for measuring the quality of service in higher education [11]. Indeed, there seems to be little consensus among researchers on how to measure the quality of teaching in higher education [12]. This work provides a method for measuring service quality in higher education, in a time of considerable upheaval for educational institutions due to COVID-19.

Service quality in higher education has been widely researched by managers and investigators due to its importance in economic terms, in reducing costs and gauging student satisfaction [11,13]. Universities need to consider service quality and students’ perceptions when designing strategies that can boost their rankings [11]. Teaching and learning in the higher education setting differ from other industries in that they cannot be separated into two parts [14]. Education and knowledge are unlike other products and services because they cannot be quantified in purely monetary terms. Higher education institutions bear the considerable responsibility of preparing students for life, not just for earning a good income [15].

In higher education, the main clients are the students [16], an idea that is by no means new, as the literature demonstrates [17,18]. Ref [19] stated that student satisfaction is the only indicator for measuring service quality in higher education. Quality of service in higher education has a significant influence on student satisfaction [20]. Growing competition among higher education institutions regionally, nationally and internationally has hastened acceptance of the notion of students as the main client [21], which means that universities need to develop the means to satisfy their students at this level of education [22,23]; and this is achieved by improving the quality of service they offer [24,25]. Online learning should keep student’s motivation and minimize their frustration with this new situation [26].

The main objective of this work is to measure the quality of the virtual teaching of subjects originally designed for the classroom training. The methodology is based on a variant of the traditional Martilla and James importance-performance analysis [27], as proposed by Picón et al. [28]. Our purpose is to identify which elements or attributes of online teaching needed to be improved and developed further. Before applying this methodology, the importance of each attribute is determined using a structural equations model based on criteria posited by Allen et al. [29]. The data were obtained from a university in southern Spain. Spain has been one of the countries hit hardest by the COVID-19 pandemic, registering more than 28,000 deaths and around 250,000 cases at the time of writing. The justification for this work is that higher education institutions and education centers whose model is based on traditional or face-to-face teaching need to gain experience in, and knowledge of, e-learning methods, and improve their online teaching models.

Based on the literature [30,31,32,33,34,35,36,37,38,39,40,41] we specify the structural equation model. On the basis of the above studies, we propose that the system characteristics affect both the effectiveness of the online teaching and the relationship with teachers and evaluation. At the same time, we propose that the effectiveness of online teaching is influenced by the relationship with teachers and evaluation. Finally, we propose that these latent variables influence the satisfaction with online teaching. The importance-performance analysis is an exploratory method and, therefore, we do not define any hypothesis.

This work differs from others in this field, firstly, because it seeks to measure quality in online teaching in undergraduate courses originally designed for traditional or on-site teaching scenarios. The impact of COVID-19 has forced institutions into a real-time restructuring of teaching methodologies and assessment systems originally conceived for classroom training. While universities adapt to these changes, both teachers and students have tried to maintain the access and capacity to acquire knowledge that students enjoyed pre-pandemic. Secondly, this work focuses on a university whose teaching was neither all online nor based on a mixed system before COVID-19, but entirely on-site. Although this university deployed a virtual platform to support face-to-face teaching, classes were nearly all taught on-site. Thirdly, the service quality of the online teaching at this university was not measured by the typical SERVQUAL and SERVPERF methods but by the importance-performance analysis proposed by Picon et al. [28], which has several additional advantages over the former. Finally, the measurement of service quality in the online teaching was not performed by consulting students of a single subject or degree course but across a range of courses, in order to reinforce the external validity and increase the statistical potential of the study.

## 2. Materials and Methods

### 2.1. Data

The data were extracted from a questionnaire for undergraduate students attending a public University, in southern Spain, which was the inclusion criteria. There were not exclusion criteria as was done in Tzeng et al. [42]. A convenience sampling was used to collect the maximum number questionaries as in Tzeng et al., Năsui et al. and Zhuo et al. [42,43,44] since their participation was voluntary [44,45]. This sampling is considered valid for this study because students are appropriate research subjects, especially if they represent a population of interest [46]. This method was used to ensure a rapid and easy data collection, since sampling units are easily accessible and furthermore, it is important to emphasize its cost effectiveness. Results will be generalized to higher education students, that is why the students were selected from a range of degree courses to reinforce external validity and to increase statistical power [30]. The questionnaire was delivered to the students by various media, firstly, via the university’s virtual platform, with the collaboration of the teachers. The survey was also sent out via the university’s Twitter account. In order to achieve the biggest response possible, the researchers contacted the delegates and sub-delegates of the various faculties who distributed the questionnaire by email and social media, as well as the student union, which sent out the survey by similar channels. The number of valid responses received was 467, some 5% of the total number of students enrolled at the university across all degree courses.

### 2.2. Instrument and Variables

The data was extracted from a structured questionnaire in which the students had to value the attributes on a scale of 0–7, in which 0 represented “of lowest value” and 7 “of highest value”. The questionnaire contained 14 items grouped as four latent variables. The four latent variables were system characteristics (CAR), effectiveness of online teaching (EFIC), relationship with teachers and evaluation (PROF), and satisfaction with online teaching (SAT). The items were selected from the literature on e-learning and quality of teaching (Table 1). Attributes representing four dimensions were analysed by means of a structural equations model.

**Figure 1:**
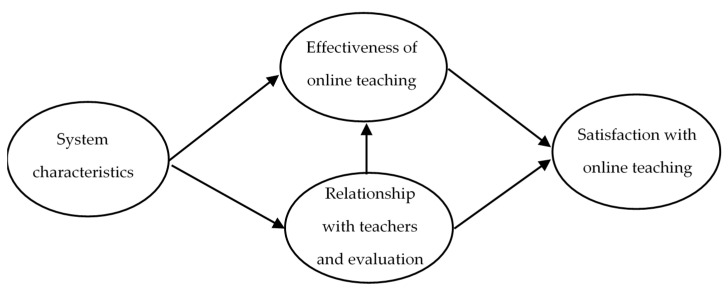
Structural model specified for the derivation of the importance of attributes.

The structural equations model was based on the literature (Figure 1). This model posits that the system’s characteristics have a direct effect on the efficacy of virtual training and on students’ relations with their teachers and aspects of the evaluation. Likewise, these latent variables have a direct effect on student satisfaction with online teaching. The structural equation model was estimated using the AMOS software (International Business Machines Corporation IBM, Armonk, NY, USA).

### 2.3. Statistical Analysis

The data were analyzed using SPSS (International Business Machines Corporation IBM, Armonk, NY, USA). The Cronbach’s Alpha coefficient for the value scores was used to determine the reliability of the scales, which came back as higher than 0.7, thus confirming the reliability of the questionnaire. To check the questionnaire’s validity, a factor analysis of the valuations was performed, using the Kaiser-Meyer-Olkin measure, which was 0.75. The null hypothesis was rejected by the Bartlett sphericity test, thus the use of the factor analysis was deemed acceptable. The factor analysis yielded four factors that represent the four dimensions in Table 1, thus confirming the validity of the questionnaire. The importance-performance analysis is required in order to know the importance of each attribute that was obtained by applying the structural equations model, based on the criteria of Allen et al. [29].

## 3. Results

The sociodemographic characteristics of the sample are showed in Table 2. We can see that most students were women, in their first year of undergraduate studies. The mean age of participants was about 21 years.

The maximum likelihood model was used to calculate the model parameters. Although the data did not comply with the multivariate normality assumption, this method facilitates the convergence of estimates even in the absence of multivariate normality [49]. Criteria in Bollen [50] and Rindskopf and Rose [51] were used to evaluate the model, in which the measurement and structural models were assessed separately. A reliability and validity analysis were performed to evaluate the measurement model. To test for reliability, an analysis of the reliability of the items and each construct was carried out. Convergent and discriminant validity were analyzed to test for validity. Results are shown in Table 3 and Table 4.

The reliability of the items was measured in order to check that the standardized load factors were higher than 0.7, in order that the variance shared between the construct and its indicator was higher than the error variance [52,53], though some authors consider a load factor above 0.5 to be acceptable [54]. Table 3 shows that all the standardized load factors exceed 0.7. The load with a value of 0.580 was considered valid, in line with criteria provided by Chau [46]. The reliability of the construct was measured by the Cronbach Alpha and composite reliability (CR) coefficients. Table 3 shows that both the Cronbach Alpha and CR coefficient values are higher than 0.7, thus, the reliability of the constructs is confirmed.

The validity of the measurement model was determined by the convergent and discriminant validity. Convergent validity was measured by the average variance extracted (AVE). Column 5 of Table 3 shows that all AVE coefficient values exceed 0.5, which verifies the convergent validity. Furthermore, for discriminant validity, all correlations between the constructs were calculated in Table 4.

Table 4 shows that these correlations were less than the AVE square root for each construct, except for the EFIC latent variable. Given that the correlation values for this variable are very similar to the AVE square root, it can be said that the discriminant validity is verified.

The structural model was assessed according to the squared correlation coefficient values and the significance of the paths or regression coefficients. Table 5 shows that all the coefficients attain a level of significance of 1%, while the squared multiple correlations are all higher than 0.3, which verifies the nomological or predictive validity. These results support the hypotheses of the model.

Finally, goodness of fit of the structural model was determined by a set of measures presented in Table 6, with reference to the absolute fit, incremental fit and parsimony of the model.

The χ^2^ statistic indicates whether the discrepancy between the original data matrix and the reproduced matrix is significant. In this case, the *p*-value indicates rejection of this hypothesis. However, the χ^2^ statistic value is strongly influenced by the sample size, the model’s complexity and the violation of the multivariate normality assumption. All the other measures also have values within the limits that allow us to confirm the data’s goodness of fit. To calculate the importance of each attribute, we applied the criteria of Allen et al. [29], according to which, importance derives from the total of the effects of each latent variable on the satisfaction with the online teaching variable (Table 7).

For calculating the relative importance of each attribute, the total effects of each latent variable were distributed among the attributes that constitute it, with the standardized regression weights also considered, as shown in Table 8. The relative importance of each attribute was calculated by multiplying the total effects of each latent variable by the standardized regression weight.

For IPA, we needed to know the values for importance and satisfaction in each attribute (Table 9). As previously mentioned, importance was determined by the structural equations model. Satisfaction was measured by the judgements expressed by the participants in the questionnaire. The values obtained show that importance ranges between 0.33 (lowest) and 0.61 (highest), while the range of satisfaction is between 0.39 (lowest) and 4.08 (highest). Nevertheless, IPA requires the values to appear on the same scale, thus, a normalization of between 0.00 and 1.00 was performed on the lowest and highest values for importance and satisfaction, respectively, to obtain the normalized values (see columns 4 and 5 of Table 8). Furthermore, column 6 of Table 9 presents the discrepancy values obtained, indicating the difference between importance and satisfaction.

**Figure 2:**
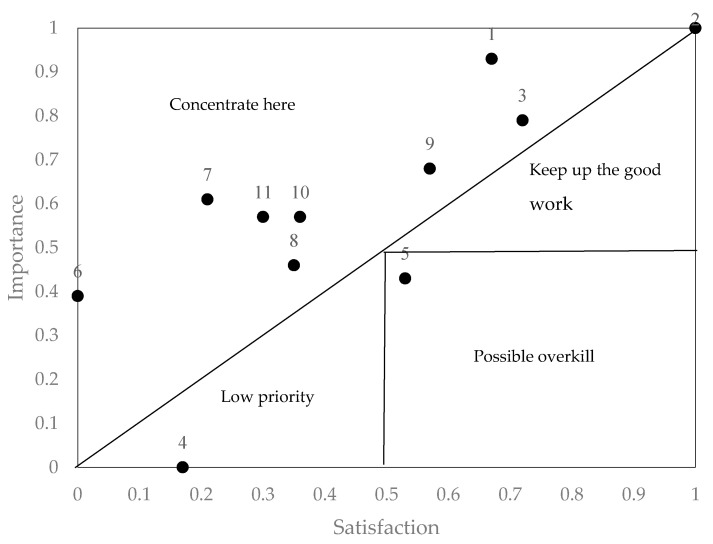
Representation of combined classic and diagonal models.

According to the values obtained in Table 9, the IPA representation was performed in Figure 2. To enhance the Martilla and James [27] representation, a combination of classic models was used in opposition to the diagonal models that divide the analysis space in two halves [28,55,56,57]. The diagonal models are based on the calculation of the discrepancies. The attributes with positive discrepancies are those in which importance exceeds satisfaction, thus, they represent a high priority for improvement. The greater the discrepancy, the more urgent the priority to attend to the attribute. In Figure 2, the attributes with a positive discrepancy are situated above the diagonal, in the quadrant named “concentrate on this”, whereas the attributes with negative discrepancies are below the diagonal. In this case, by combining the diagonal models with the classic Martilla and James [27] representation we differentiate three areas in which, based on the values of importance and satisfaction, we identify those attributes that indicate continuing with good work, attributes of low priority and attributes of a potential waste of resources.

**Figure 3:**
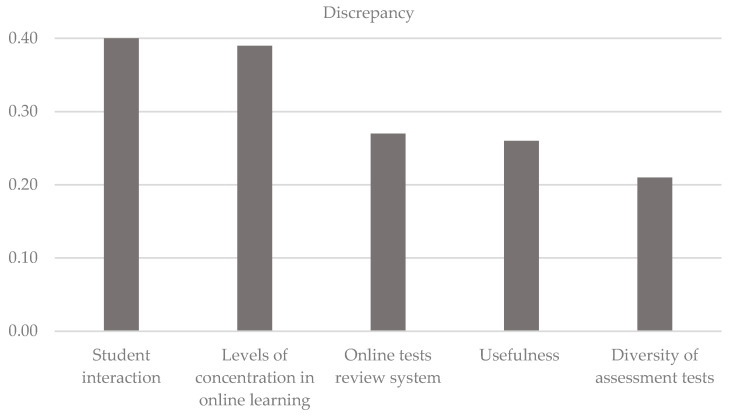
Representation of the five priority attributes.

It can be observed that there are eight attributes that fall within the “concentrate on this” area, meaning that these attributes need to be improved. The attributes that require attention are those that are furthest from the diagonal. In this case, the discrepancies show that the attributes most in need of improvement are interaction with other students (attribute 7), levels of concentration in online classes (attribute 6), online tests review system (attribute 11), usefulness of the system (attribute 1) and diversity of activities used in student assessment (attribute 10). There is no attribute in the “continue with the good work” quadrant, while the “low priority” quadrant contains learning speed in online teaching compared to face-to-face teaching (attribute 4). The possible waste of resources quadrant has the online learning autonomy attribute. The results clearly show that there is a general level of dissatisfaction among students with the online teaching rolled out since the outset of COVID-19. Finally, Figure 3 shows the five priority attributes of online teaching that need to be improved.

## 4. Discussion

Universities need to adapt to the new situation created by the COVID-19 pandemic. They need to convert face-to-face teaching into an online format that is generally acceptable by their students. Integrating methodological and technological decisions should not just be a task for the higher education institutions as entities but should also involve collaboration among professors, students and administration staff and services. The results of this study suggest a set of priority areas that require attention in order to improve student satisfaction with online training. The first is interaction between students, as the attribute that presented the highest level of discrepancy. Following Ferri et al. [58], the lack of interactivity is one of the main pedagogical challenges associated with online teaching. In spite of online teaching, Verawardina et al. [59] hold that it is necessary maintain the interaction of direct contacts with other humans. Student interaction may have a decisive influence on the achievement of educational goals [60]. Such interaction may be highly beneficial for students especially in learning situations where moderately divergent points of view can be debated. However, virtual training can leave students feeling lonely and isolated, and reduce their capacity to form interpersonal relationships and accrue the consequent educational benefits. Nevertheless, online interactions can be useful and advantageous for students, especially through good group activity planning or conscientious teacher performance.

The second attribute that requires attention is the level of student concentration in online classes. One of the main problems that students face with virtual training is distraction; concentration wavers when sat in front of a computer screen for a length of time. Distraction can take the form of fatigue, email notifications, social network activity, phone calls, unexpected home visits, etc., all of which reduce student productivity and concentration during online classes. To improve student concentration, online sessions could be shorter, mind breaks could be inserted into the planning, along with mini-tasks or short activities. Students’ attention may also be reduced if they have other open apps on the screen during the online lessons. To resolve this, there are apps that can be activated by the institution to block access to other apps not related to the work being done by students in real time online classes.

The third attribute that requires improvement in order to raise student satisfaction with virtual learning is the system for reviewing online tests. This is also related to the problematic question of online assessments, in particular the need to know the identity of the student doing the test and to ensure that it is genuine, control over the physical context in which the assessment test takes place, and the relevant data protection legal framework. Many educational institutions are striving to overcome these problems using e-proctoring systems [61]. However, as García-Peñalvo et al. [62] indicate, assessment is a complex process that needs to be performed throughout a fixed instruction/learning period, not just at specific moments during the course. In most cases, the design of assessment test reviews in the virtual model is similar to those in the face-to-face teaching model, in other words, it is limited to indicating correct and incorrect answers, but now online instead of face-to-face. The problems that students perceive in this attribute, and the diversity of assessment tests attribute, could be the cause of their general dissatisfaction with online learning.

The fourth attribute that requires attention in order to improve students’ satisfaction with online teaching is the usefulness of the system. When the virtual instruction system functions well, students save time in the learning process, it boosts their sense of learning self-sufficiency and can improve results [31,32,33,63]. One possible reason for student dissatisfaction with how the system works is their lack of experience in managing it. This study has emphasized how the pandemic forced live classes to shift suddenly to the virtual format. A system’s usefulness is greatly affected by the technological media available both to the educational institutions and to the users. This highlights the need to improve the systems for imparting virtual instruction and to address the digital divide among users [64].

The fifth attribute to be tackled is the diversity of assessment tests. The lockdowns ordered as a result of the pandemic obliged the majority of education centers to close and switch rapidly from a face-to-face teaching design to one that could be implemented online. Assessment tests have continued to be set, and adapted to virtual learning, but changing the format. Now, however, teachers need to take advantage of the range of assessment activities available online to broaden the range of testing formats, and to increase student satisfaction with the assessment system. The remaining attributes that educational institutions need to attend in order to enhance student satisfaction are of a lesser priority than those already discussed. One such low-priority attribute is the learning speed in online teaching compared to face-to-face teaching. This attribute has a negative discrepancy but low importance among students. This could be due to the fact that students’ knowledge acquisition is a semester-long process because the university courses for which data are available are taught in six-monthly segments, hence the very low importance students attach to this attribute.

Online learning autonomy is also an attribute of little importance to the students, although the level of satisfaction is high, thus, it is situated in the quadrant denoting possible waste of resources. Learning autonomy refers to students’ gradual acquisition of their own criteria, methods and rules for transforming them into more effective learners. In online instruction, students establish their own pace of learning by accessing content anytime and as often as is necessary for them to acquire knowledge [65]. This could explain the low importance, but high level of satisfaction attached to this attribute.

## 5. Conclusions

This work emphasizes the enormous efforts made by educational institutions to transform content, activities and assessment systems designed for face-to-face teaching to online formats. This was done urgently, and often without the main actors possessing the minimum technological media, nor the digital competences and aptitudes required to adapt to such rapid change [66]. The results of this study show a general dissatisfaction among students with many of the features of online teaching that have become apparent in the period of lockdown in Spain due to COVID-19. The results do not apply to educational institutions that exclusively teach courses online, but to those institutions that had to adapt quickly, and shift course material originally designed for face-to-face instruction to the online format without enough time to carry out a more thorough process of adaptation.

This work contributes to the generation of knowledge about online teaching. The results provide valuable information for education professionals and educational institutions. The challenges posed by online teaching should be more related to the effectiveness of learning and human relations than to the technical characteristics of the systems used. Aspects such as improving interaction between students or between student/teacher, improving concentration or aspects of assessment must be taken into account in order to improve student satisfaction.

The situation with COVID-19 should not be seen as a threat by students and educational institutions based on conventional teaching but should serve to adapt the benefits of the online education system to their teaching.

At the societal level, online education also has important implications. Aspects such as travel costs and time savings are drastically reduced. On the other hand, for online learning to be effective, students must have reliable access to technology. This requires the development of social and economic policies to break the digital divide in many households and improve access to technology.

Like many other investigations in this field, our study has limitations. Although the results of this work can be extrapolated to other educational institutions that have undergone similar changes, the data for this study are based on a single Spanish university. Future lines of investigation would need to compare the data in our study against other universities, both inside and outside Spain. It would also be interesting to study whether differences exist between the levels of student satisfaction with online instruction in terms of course content and areas of knowledge.

## Figures and Tables

**Table 1:** Dimensions and attributes selected to measure quality.

	Dimension	Variable	Attributes
CAR	System characteristics	CAR1	Usefulness [31,32,33]
		CAR2	Easy to use [32,34,35,36]
		CAR3	Technical support availability [37,38]
EFIC	Efficacy of online teaching	EFIC1	Quick learning versus face-to-face teaching [32]
		EFIC2	Autonomy in online learning [31,32]
		EFIC3	Concentration in online classes [34,39]
		EFIC4	Interaction with other students [39,40,41]
PROF	The students’ relationships with their professors and the evaluation of the professors	PROF1	Interaction with the professors [39,40,41]
		PROF2	Professors’ response time [32]
		PROF3	Diversity of assessment activities [47]
		PROF4	Online test review system [48]
SAT	Satisfaction with online teaching	SAT1	I am satisfied with online teaching as a learning method [32,41]
		SAT2	I am satisfied with online teaching [32].
		SAT3	Online teaching satisfies my learning needs [41].

**Table 2:** Sociodemographic characteristics of the sample.

Gender	**Men**		**Women**	
	37.9%		62.1%	
Year of undergraduate studies	**First**	**Second**	**Third**	**Fourth**
	34.1%	26.4%	20.9%	18.6%
Statistics	**Mean**		**Standard Deviation**	
	21.34 years		2.45 years	

**Table 3:** Standardized estimations for observable indicators, Cronbach’s α values, convergent validity, and reliability assessment.

Factors			λ	Cronbach’s α	Composite Reliability (CR)	AVE
System characteristics				0.896	0.897	0.744
CAR1	←	CAR	0.879			
CAR2	←	CAR	0.896			
CAR3	←	CAR	0.812			
Efficacy of online teaching				0.851	0.853	0.596
EFIC1	←	EFIC	0.580			
EFIC2	←	EFIC	0.801			
EFIC3	←	EFIC	0.785			
EFIC4	←	EFIC	0.890			
Teacher performance and assessment characteristics				0.884	0.881	0.650
PROF1	←	PROF	0.757			
PROF2	←	PROF	0.859			
PROF3	←	PROF	0.807			
PROF4	←	PROF	0.801			
Student satisfaction with online teaching				0.952	0.952	0.868
SAT1	←	SAT	0.919			
SAT2	←	SAT	0.925			
SAT3	←	SAT	0.951			

**Table 4:** Discriminant validity of measures.

	CAR	EFIC	PROF	SAT
CAR	0.863			
EFIC	0.768	0.772		
PROF	0.793	0.808	0.807	
SAT	0.675	0.811	0.762	0.932

**Table 5:** Parameter estimates.

Relation			Estimate	S.E.	C.R.	Standardized Estimate	*p*
PROF	←	CAR	0.617	0.040	15.465	0.793	***
EFIC	←	CAR	0.204	0.040	5.097	0.343	***
EFIC	←	PROF	0.409	0.060	6.873	0.536	***
SAT	←	EFIC	0.859	0.115	7.495	0.563	***
SAT	←	PROF	0.358	0.076	4.702	0.307	***
CAR1	←	CAR	1.000			0.879	
CAR2	←	CAR	1.024	0.039	25.985	0.896	***
CAR3	←	CAR	0.886	0.040	22.139	0.812	***
EFIC1	←	EFIC	1.000			0.580	
EFIC2	←	EFIC	1.338	0.103	12.966	0.801	***
EFIC3	←	EFIC	1.468	0.115	12.804	0.785	***
EFIC4	←	EFIC	1.485	0.108	13.735	0.890	***
PROF1	←	PROF	1.000			0.757	
PROF2	←	PROF	1.135	0.059	19.192	0.859	***
PROF3	←	PROF	1.135	0.063	17.925	0.807	***
PROF4	←	PROF	1.074	0.060	17.840	0.801	***
SAT1	←	SAT	1.000			0.919	
SAT2	←	SAT	1.036	0.030	34.881	0.925	***
SAT3	←	SAT	1.014	0.027	37.779	0.951	***

*** *p* < 0.001.

**Table 6:** Goodness of fit of the structural model.

χ^2^	df	RMSEA	NFI	NNFI	CFI	GFI	AGFI	PNFI
335.886 *	1.951	0.063	0.917	0.951	0.957	0.866	0.832	0.797

Note: * *p*-value < 0.05; df = degrees of freedom; RMSEA: root mean square error of approximation; NFI: normed fit index; NNFI: non-normed fit index; CFI: comparative fit index; GFI: goodness of fit index; AGFI: adjusted goodness of fit index; PNFI: parsimony normed fit index.

**Table 7:** Effects of predictor variables.

	Direct Effect	Indirect Effect	Total Effect
CAR	0.000	0.675	0.675
EFIC	0.563	0.000	0.563
PROF	0.307	0.302	0.609

**Table 8:** Calculation of importance.

Attribute	Total Effect	Standarized Coefficients	Importance
Usefulness	0.675	0.879	0.59
Ease-of-use		0.896	0.61
Support availability		0.812	0.55
Learning speed in online vs. face-to-face teaching Online learning autonomy Levels of concentration in online learning Student interaction	0.563	0.580	0.33
		0.801	0.45
		0.785	0.44
		0.890	0.50
Student interaction with teachers Teacher response times Diversity of assessment tests Online tests review system	0.609	0.757	0.46
		0.859	0.52
		0.807	0.49
		0.801	0.49

**Table 9:** Importance, satisfaction and discrepancies.

#	Attribute	Importance	Satisfaction	Normalized Importance	Normalized Satisfaction	Discrepancies
1	Usefulness	0.59	3.49	0.93	0.67	0.26
2	Ease-of-use	0.61	4.08	1.00	1.00	0.00
3	Support availability	0.55	3.59	0.79	0.72	0.07
4	Learning speed in online vs. face-to-face teaching	0.33	2.62	0.00	0.17	-0.17
5	Online learning autonomy	0.45	3.26	0.43	0.53	-0.10
6	Levels of concentration in online learning	0.44	2.32	0.39	0.00	0.39
7	Student interaction	0.50	2.69	0.61	0.21	0.40
8	Student interaction with teachers	0.46	2.93	0.46	0.35	0.11
9	Teacher response times	0.52	3.33	0.68	0.57	0.11
10	Diversity of assessment tests	0.49	2.96	0.57	0.36	0.21
11	Online tests review system	0.49	2.85	0.57	0.30	0.27

## Data Availability

The data presented in this study are available on request from the corresponding author. The data are not publicly available due to privacy or ethical restrictions.
